# A Case Report of Disseminated Blastomycosis With Thyroid Involvement in a Pregnant Patient

**DOI:** 10.7759/cureus.8508

**Published:** 2020-06-08

**Authors:** Amit L Jain, Rahul Peravali, Hemnishil K Marella, Reshma Premkumar, Ankur Seth

**Affiliations:** 1 Internal Medicine, University of Tennessee Health Science Center, Memphis, USA; 2 Medicine, University of Tennessee Health Science Center, Memphis, USA

**Keywords:** blastomyces dermatitidis, blastomycosis, disseminated blastomycosis, thyroid

## Abstract

*Blastomyces dermatitidis *is the causal agent of blastomycosis, an invasive and often serious fungal infection. Blastomycosis typically presents as a pulmonary infection, but common extrapulmonary manifestations of blastomycosis include the skin, bones, and reticuloendothelial systems. Disseminated blastomycosis occurs more prominently in immunocompromised individuals, such as organ transplant recipients, HIV patients, and pregnant women. We report here a rare case of disseminated blastomycosis to the thyroid in a pregnant patient. This case emphasizes the unique challenges of diagnosing and treating disseminated fungal infections in pregnancy.

## Introduction

Blastomycosis is a systemic granulomatous infection caused by the dimorphic fungus *Blastomyces dermatitidis *(*B. dermatitidis*). Blastomycosis is endemic to the eastern United States, especially the Ohio and Mississippi River Valleys, the Great Lakes, and St. Laurence Rivers [1]. The infection is transmitted by inhaling decomposing wood or vegetation and often presents clinically as acute pneumonia, chronic pneumonia, or an asymptomatic radiographic abnormality on chest x-ray [2]. Although blastomycosis typically presents as a pulmonary infection, extrapulmonary blastomycosis has been reported in as many as 25%-40% of cases [3]. Common extrapulmonary manifestations of blastomycosis include the skin, bones, central nervous system, genitourinary, and reticuloendothelial systems [3]. We present here a rare case of disseminated blastomycosis to the thyroid diagnosed by thyroid fine-needle aspiration (FNA) in a pregnant patient. 

## Case presentation

A 21-year-old G1P0 at 29 weeks' pregnancy with a past medical history of asthma presented with a two-day history of productive cough, chills, shortness of breath, and high-grade fevers. Initial workup was notable for positive rapid influenza, and a left mid and lower quadrant consolidation on chest x-ray concerning for pneumonia. She received intravenous fluids, azithromycin 500 mg, ceftriaxone 1 g, and oseltamivir phosphate 75 mg. She denied any history of alcohol intake and smoking cigarettes. 

On presentation, vital signs were significant for a low-grade fever of 100.2 degrees Fahrenheit, a heart rate of 140 beats/minute, a respiratory rate of 40 breaths/minute, a blood pressure of 97/51 mmHg, and oxygen saturation of 96% on room air. Physical examination was notable for decreased breath sounds bilaterally on lung exam and a tender, fluctuating, round lesion consistent with a subcutaneous abscess on the left inner thigh with a small area of surrounding cellulitis. Initial labs were significant for a white blood cell (WBC) count of 37 K/uL, a hemoglobin of 6.9 g/dL, and hypoglycemia with low glucose of 60 mg/dL. The patient was admitted to the telemetry floor, and antibiotics were continued. 

**Figure 1:**
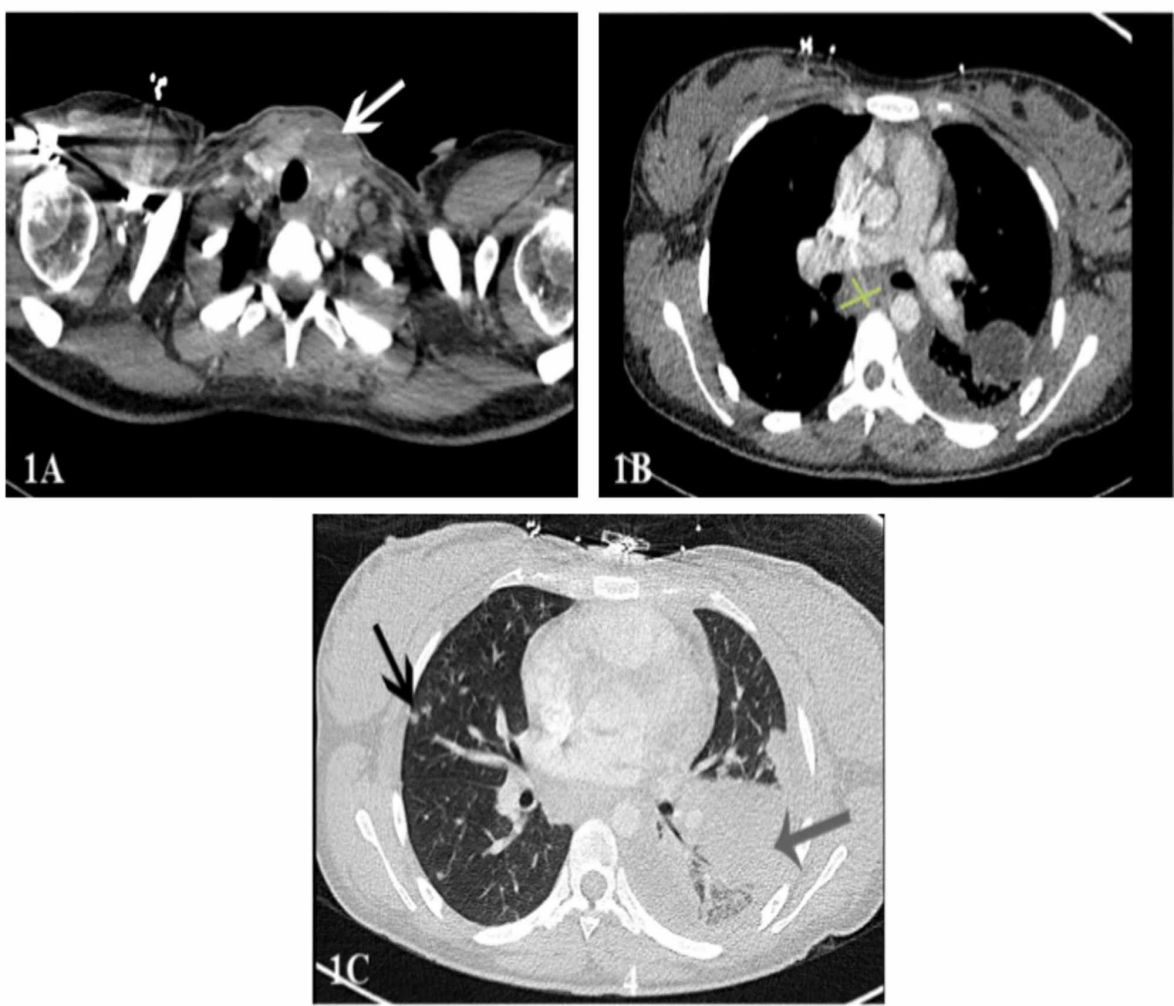
Initial CT chest with contrast (A) A 3.2 cm x 2.4 cm mass-like density within the left thyroid lobe. (B) Multiple mediastinal lymph nodes with the largest being subcarinal measuring approximately 1.7 cm x 3.6 cm. (C) Left lower lobe consolidation and a small pleural effusion (gray arrow). Additionally, throughout bilateral lungs, there are many subcentimeter solid noncalcified nodules (black arrow).

After two days of adequate community-acquired pneumonia coverage (ceftriaxone and azithromycin), the patient's respiratory status continued to decline and hence was transferred to the ICU. The patient's WBC count continued to remain elevated at 40 K/uL. A repeat chest x-ray revealed a large left-sided pleural effusion. A diagnostic and therapeutic thoracentesis of 600 mL was performed. Pleural studies were consistent with an uncomplicated parapneumonic effusion. The antibiotics were broadened by the infectious disease (ID) team to vancomycin and meropenem, and azithromycin was continued. After a few days of the antibiotics, the patient's respiratory status was stable but continued to have elevated WBC. A computed tomography (CT) chest with contrast was performed, which showed left lower lobe pneumonia, a 3.2 x 2.4 mass-like density within the left thyroid lobe, innumerable solid noncalcified nodules throughout bilateral lungs, and multiple enlarged mediastinal lymph nodes (Figure 1).

**Figure 2:**
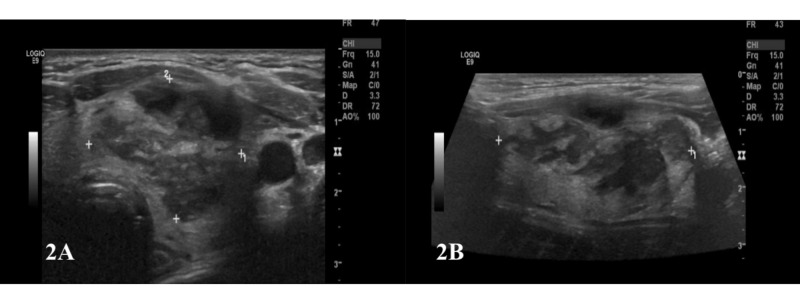
Thyroid ultrasound Images A and B showing an ill-defined 2.7 x 2 x 4.3 cm heterogeneous mass occupying most of the left lobe.

Thyroid ultrasound further characterized the unusual thyroid finding as an ill-defined 2.7 x 2.0 x 4.3 cm heterogeneous mass occupying most of the left lobe of the thyroid (Figure 2). Follow-up thyroid studies were within normal limits. 

**Figure 3:**
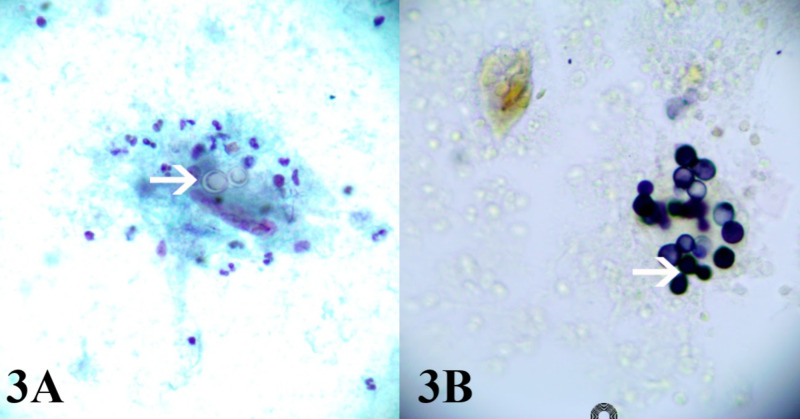
Left thyroid fine-needle aspiration biopsy (A) *Blastomyces dermatitidis* organism yeast cell with thick double refractive walls. (B) Gomori methenamine silver stain performed on a cytospin slide highlights fungal organism broad-based budding cells morphologically consistent with *B. dermatitidis*.

**Figure 4:**
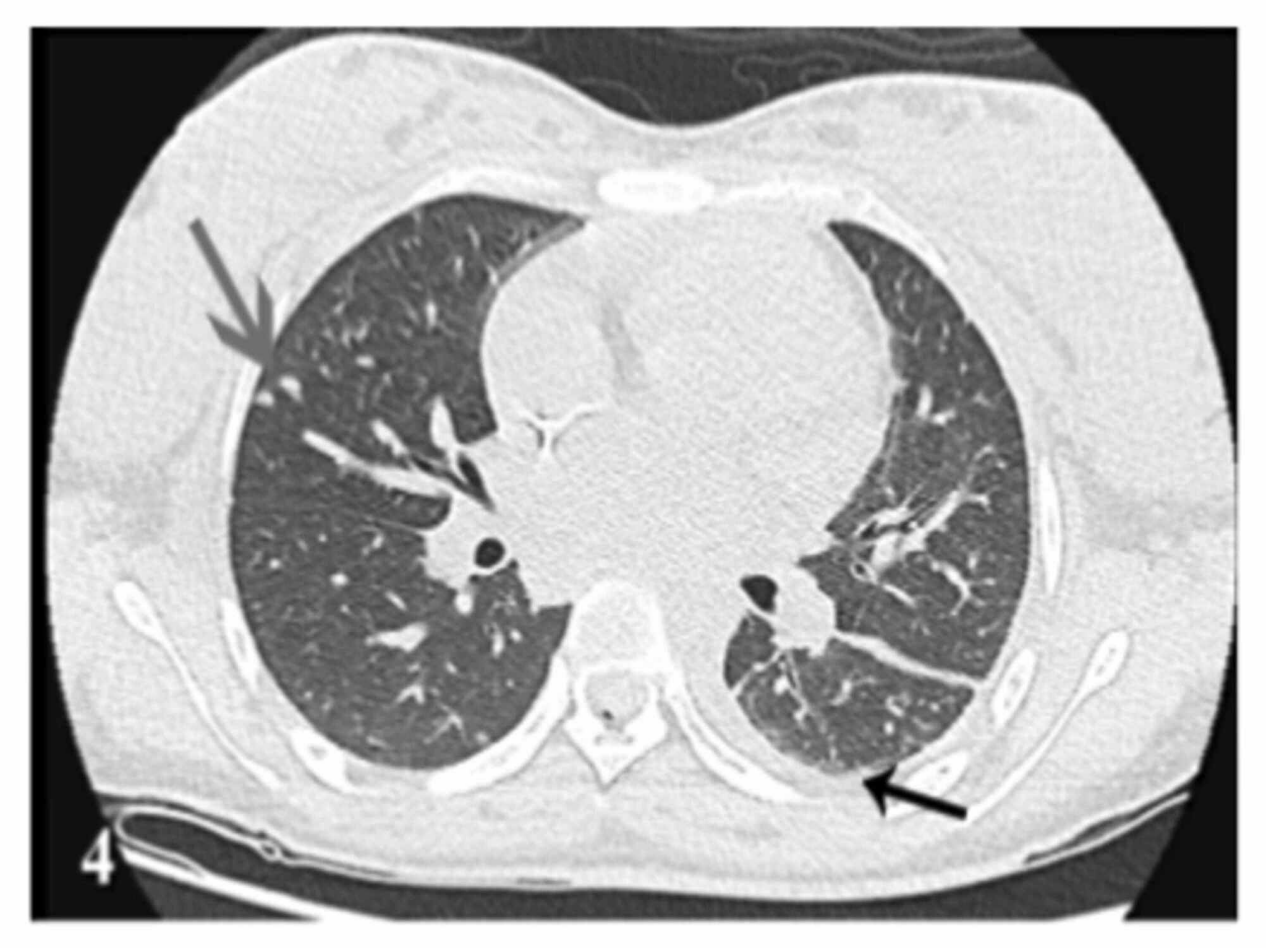
Follow-up CT chest without contrast Follow-up CT chest without contrast after two weeks showing improving pneumonia (black arrow). Also, many solid noncalcified nodules decreased in size (gray arrow).

The autoimmune workup was negative. Fungal workup was positive for histoplasmosis and blastomycosis serum antigen, but a negative Fungitell test (Associates of Cape Cod, East Falmouth, MA). ID team determined that the patient's presentation was most consistent with blastomycosis infection. Given the patient's pregnancy status, mediastinal lymphadenopathy, and thyroid nodule, disseminated blastomycosis was suspected. ID team initiated liposomal amphotericin B (5 mg/kg), on which the patient improved remarkably. Once the patient became medically stable, ultrasound-guided FNA and Gomori methenamine silver (GMS) stain were performed, which showed fungal organisms morphologically consistent with *B. dermatitidis*. (Figure 3). After two weeks of amphotericin B treatment, a repeat CT chest without contrast showed improving pneumonia (Figure 4).

 

Of note, the patient was persistently hypoglycemic in the ICU and required a dextrose 10% drip. It was suspected that her hypoglycemia was secondary to a combination of the hypermetabolism of pregnancy and disseminated fungal infection. High-risk obstetrics team was consulted, who recommended that the baby be delivered at 32 weeks. The patient was induced and had an uncomplicated delivery and post-partum hospital stay. We discharged the patient on itraconazole therapy 200 mg twice a day for one year per ID team recommendation. We also recommended the patient's baby to be treated empirically for blastomycosis. 

## Discussion

Blastomycosis typically presents as a pulmonary infection. However, disseminated blastomycosis can have extrapulmonary manifestations, especially involving the skin, bones, and reticuloendothelial systems. Disseminated blastomycosis occurs more prominently in immunocompromised individuals, such as organ transplant recipients, HIV patients, and pregnant women [1]. We present here a rare case of disseminated blastomycosis to the thyroid diagnosed by thyroid FNA in a pregnant patient. To our knowledge, this is the first case of thyroid blastomycosis in a pregnant woman.

Thyroid blastomycosis is a rare phenomenon that has been reported in only a handful of cases in the literature [4-7]. In contrast to most other organs in the body, the thyroid gland is remarkably resistant to infection. The thyroid's rich lymphovascular network, protective fibrous capsule, and high iodine content make it difficult for fungal organisms to invade and colonize it [8]. Many patients with disseminated fungal infections to the thyroid often show no clinical manifestations of thyroiditis [8]. FNA of the thyroid in patients with a high suspicion of disseminated fungal infection can provide a quick and reliable diagnosis. Blastomycosis, for example, has a highly characteristic "broad-based bud" morphology under cytology [2].

Disseminated fungal infection in pregnancy poses a unique threat to both mother and baby. Prompt disease recognition and timely initiation of antifungal therapy in pregnancy can halt further progression and prevent possible transplacental dissemination. Liposomal amphotericin B followed by oral itraconazole is the treatment of choice for disseminated extrapulmonary blastomycosis in nonpregnant patients [1]. However, little is known regarding optimal antifungal regimens and dosages in pregnancy [9]. Antifungal therapy in pregnancy requires a careful consideration of maternal benefits and fetal risks, including fetal loss, congenital malformations, and prematurity [9]. Azoles, in particular, should be avoided because of possible teratogenicity [1]. When appropriate, early delivery of the baby should also be considered. 

## Conclusions

This case study presents a unique case of disseminated thyroid blastomycosis in a pregnant woman. Clinicians need to recognize the vast array of clinical presentations of disseminated blastomycosis, especially in immunocompromised patients. A high level of suspicion in endemic areas can expedite early diagnosis and facilitate appropriate treatment. 

## References

[1] Chapman SW, Dismukes WE, Proia LA (2008). Clinical practice guidelines for the management of blastomycosis: 2008 update by the Infectious Diseases Society of America. Clin Infect Dis.

[2] Saccente M, Woods GL (2010). Clinical and laboratory update on blastomycosis. Clin Microbiol Rev.

[3] Smith JA, Kauffman CA (2010). Blastomycosis. Proc Am Thorac Soc.

[4] Moinuddin S, Barazi H, Moinuddin M (2008). Acute blastomycosis thyroiditis. Thyroid.

[5] Wineland A, Siegel E, Francis C, Chen C, Bodenner D, Stack BC (2008). Fine-needle aspiration diagnosis of thyroid blastomycosis. Endocr Pract.

[6] Harvery AM, Mody DR, Amrikachi M (2011). Disseminated blastomycosis diagnosed by fine-needle aspiration of the thyroid. Diagn Cytopathol.

[7] Rao N, Mann SJ (2017). Fine needle aspiration cytology of acute blastomycosis thyroiditis. Diagn Cytopathol.

[8] Goldani LZ, Zavascki AP, Maia AL (2006). Fungal thyroiditis: an overview. Mycopathologia.

[9] Pilmis B, Jullien V, Sobel J, Lecuit M, Lortholary O, Charlier C (2014). Antifungal drugs during pregnancy: an updated review. J Antimicrob Chemother.

